# Nonlinear radiation effect on MHD Carreau nanofluid flow over a radially stretching surface with zero mass flux at the surface

**DOI:** 10.1038/s41598-018-22000-w

**Published:** 2018-02-27

**Authors:** Dianchen Lu, M. Ramzan, Noor ul Huda, Jae Dong Chung, Umer Farooq

**Affiliations:** 1 Faculty of Science, Jiangsu University, Zhenjiang, Jiangsu China; 2 Department of Computer Science, Bahria University, Islamabad Campus, Islamabad, 44000 Pakistan; 3 Department of Mathematics, Quaid-i-Azam University, Islamabad, Pakistan; 4 Department of Mechanical Engineering, Sejong University, Seoul, 143-747 Korea; 5 Department of Mathematics, COMSATS Institute of Information Technology, Park road, Tarlai Kalan, Islamabad, 45550 Pakistan

## Abstract

A mathematical model is envisaged to study the axisymmetric steady magnetohydrodynamic (MHD) Carreau nanofluid flow under the influence of nonlinear thermal radiation and chemical reaction past a radially stretched surface. Impact of heat generation/absorption with newly introduced zero mass flux condition of nanoparticles at the sheet is an added feature towards novelty of the problem. Further, for nanofluid the most recently organized model namely Buongiorno’s model is assumed that comprises the effects thermophoresis and Brownian motion. Utilizing suitable self-similar transformations, the set of partial differential equations with high nonlinearity are converted into a dimensionless system of ordinary differential equations. Set of these transmuted equations are numerically solved by MATLAB built-in function bvp4c. Impact of germane parameters on all involved profiles are plotted to examine the heat and mass transfer characteristics. This study reveals that the temperature distribution is an escalating function of the heat generation and nonlinear radiation parameters. Also, it is noted that the incrementing values of chemical reaction parameter lowers the nanoparticles concentration profile. A comparison of the present investigation with already published explorations in limiting case is also added to authenticate the presented results; hence reliable results are being presented.

## Introduction

Fluids used in industry are vital because of their ability to increasing/decreasing energy discharge to the systems. In advanced manufacturing and thermal processes characteristics like thermal conductivity, heat capacity and other physical features are core ingredients of such effects. Heat transfer efficiency in manufacturing processes may be affected if thermal conductivity is poor. Excellent heat transfer capability is core requirement in numerous industrial and engineering processes like power generation, polymer extrusion processes, paper production, chemical processes and glass fiber etc. Enhancement in thermal conductivity may be achieved by adding suspended metallic particles in the industrial liquids. Nanofluids^1^ are mixture of metallic particles submerged in base fluids which are generally of low thermal conductivity. These are modern heat transfer agents that trigger the thermal conductivity of the base liquids and an essential topic for researchers and scientists over the past few years because of its varied engineering and industrial applications. Authors on their theoretical assumptions and experimental analysis have presented varied nanofluid mathematical models to boost the thermal conductivity of varied base liquids. Buongiorno^2^ presented the transport characteristics of nanofluids. He established a mechanism named as seven slip mechanism which assembles the velocity of the base fluid and nanoparticles in a parallel manner. Furthermore, he deduced that thermophoresis and Brownian diffusion are two influential slip mechanisms in nanoliquids. In the recent years, numerous investigations were communicated by several authors based on the idea of nanofluids. These include study by Minakov *et al*.^3^. who conducted an experiment to study the boiling debacle of nanofluids on cylindrical heaters. They used suspended nanoparticles of silicon, iron oxides, aluminum and diamond into a distilled water and reported that use of nanofluids provides an increase of critical heat flux. Carreau nanofluid flow in attendance of a convective boundary condition is studied by Hayat *et al*.^4^. Khan and Azam^5^ explored the heat and mass transfer mechanism on time dependent MHD flow of Carreau nanofluid. They utilized Buongiorno’s model to comprehend the upshots of Brownian motion and thermophoresis. Sulochana *et al*.^6^. scrutinized the flow of Carreau nanofluid near a stagnation point with transpiration effect within the sight of “Brownian motion and thermophoresis”. Recently, Azam *et al*.^7^. probed the unsteady radiative stagnation point flow of MHD Carreau nanofluid past an expanding/contracting cylinder. Some recent studies featuring nanofluids may be found at^8–21^ and many therein.

In the process of thermal radiation, heat energy is emitted from a radiated surface in the form of electromagnetic waves in all directions. The radiative heat transfer mechanism is the only tool for heat transfer whenever a vacuum is present. This phenomenon has a significant effect on the high temperature creation. In the areas of engineering and physics thermal radiation has a decisive effect on heat transfer and flows of different liquids. Moreover, consequence of thermal radiation has a pivotal role in space technology where immense thermal efficiency of the devices is accomplished that are being operated at extremely high temperature levels. Some recent investigations highlighting impacts of thermal radiation include investigation by Kothandapani and Prakash^22^ who discussed the motion of peristaltic MHD Williamson nanofluids under the impact of thermal radiation parameter through a tapering asymmetric channel. Williamson fluid with suspended particles accompanied by nonlinear thermal radiation effect past a stretched surface was presented by Kumar *et al*.^23^. Khan *et al*.^24^. addressed the effect of nonlinear radiation on MHD flow of Carreau nanofluid past a surface which is convectively heated. Waqas *et al*.^25^. numerically discussed mathematical model comprises of magneto Carreau nanofluid with impact of thermal radiation. The combined effects of thermal stratification and radiation on tangent hyperbolic fluid flow past flat surface and cylindrical was conversed by Rehman *et al*.^26^. Mushtaq *et al*.^27^. numerically deliberated the nanofluid flow due to solar energy with impact of nonlinear thermal radiation. Rehman and Eltayeb^28^ discussed the hydromagnetic nanofluid over a nonlinear stretched surface accompanied by convective boundary condition and thermal radiation. The impacts of chemical reaction and newtonian heating on radiative flow of Carreau liquid was deliberated by Hayat *et al*.^29^. Ramzan *et al*.^30^. addressed the impacts of nonlinear radiation and variable thermal conductivity on the flow past an Eyring Powell nanofluid in the attendance of chemical reaction.

In recent days, the problems aiming at the flow because of radially stretching sheet have attracted the interests of lots of scientists. A considerable amount of work has been done on the problems of fluid flow due to radially stretched surfaces. Amongst these, Makinde *et al*.^31^. probed the problem of variable viscosity MHD nanofluid flow past a convective radially stretching surface with effects of thermal radiation. Ahmad *et al*.^32^. perceived series solution of time dependent axisymmetric second grade fluid flow problem past a radially stretched surface. Time dependent axisymmetric flow and heat transfer was addressed by Shahzad *et al*.^33^. Weidman^34^ premeditated the rotational axisymmetric stagnation point flow affected by a radially stretched surface. Khan *et al*.^35,36^. discussed axisymmetric flow owning to radially stretched sheet. The analytic solution over a stretching sheet of axisymmetric second grade fluid flow and heat transfer is given by Hayat *et al*.^37^. Some recent attempts in this regard may be seen at^38,39^.

Heat generation/absorption process has a pivotal role in cooling process. Precise modeling of heat generation/absorption is extremely arduous; however, some straightforward mathematical models can convey its normal stance in many physical circumstances. Upreti *et al*.^40^. debated the flow of MHD Ag-water nanofluid past a flat permeable plate within the sight of suction/injection, viscous Ohmic-dissipation and heat generation and absorption. Ramzan *et al*.^41^. envisaged a model to investigate the impact of solutal and thermal stratification on Jeffery magneto-nanofluid along a slanted stretched cylinder within the sight of heat generation/absorption and thermal radiation. Unsteady flow of Falkner-Skan Carreau nanofluid past a wedge with heat generation/absorption and melting effects was dissected by Khan *et al*.^42^.

In the above literature survey, none of the problem have explored the combined impacts of nonlinear thermal radiation and heat generation/absorption on the MHD Carreau nanofluid flow past a radially stretched surface with chemical reaction. Moreover, the heat and mass transfer mechanism are studied by utilizing the boundary condition of zero-mass flux at the surface. Partial differential equations with high nonlinearity are transformed into a set of ordinary differential equations via apposite transformations. Numerical solutions are attained for the velocity, temperature and nanoparticle concentration profiles by using the MATLAB tool bvp4c. This study emphasizes the impression of nonlinear radiation parameter on “temperature and nanoparticles concentration” distributions. Additionally, the impacts of some relevant parameters, for instance, chemical reaction parameter, temperature ratio parameter and heat generation/absorption parameter on the flow and heat transfer properties are also explored through graphical and tabular aids. A comparison with a previously done exploration is also added to the present study and excellent concurrence is obtained; hence dependable results are being presented.

## Governing Equations

The basic equations for an incompressible fluid representing mass, linear momentum, energy and concentration without body forces are given as below:

1 $${\rm{div}}\,{\rm{V}}=0,$$

2 $$\rho ({\bf{V}}.{\boldsymbol{\nabla }})={\boldsymbol{\nabla }}.\,{\boldsymbol{\tau }}+{\bf{j}}\times {\bf{B}},$$

3 $$\rho c({\bf{V}}.\nabla {\rm{T}})=\nabla .k\nabla {\rm{T}}+{\rho }_{p}{c}_{p}({D}_{B}\nabla \varphi .\nabla T+{D}_{T}\frac{\nabla T.\nabla T}{{T}_{\infty }}),$$

4 $${\bf{V}}.\nabla {\rm{C}}=\nabla .({D}_{B}\nabla C+{D}_{T}\frac{\nabla T}{{T}_{\infty }}),$$

is the heat flux. Here, ***T*** *and* ***C*** are the temperature and concentration of the fluid respectively. Also, ***V***, *k*, *ρc*, *c*_*p*_, *ρ*_*p*_*c*_*p*_ and $\frac{{\bf{d}}}{{\boldsymbol{dt}}}$ are the vector field, thermal conductivity, heat capacity of nanofluid, specific heat, heat capacity of nanoparticles and time derivatives respectively.

The Cauchy stress tensor ***τ*** modeled for Carreau rheological model is given by:

5 $${\boldsymbol{\tau }}=-{\rm{p}}{\bf{I}}+{\rm{\mu }}{{\bf{A}}}_{1},$$

with

6 $${\rm{\mu }}={{\rm{\mu }}}_{\infty }+({{\rm{\mu }}}_{0}-{{\rm{\mu }}}_{\infty }){[1+{{\rm{\mu }}}_{\infty }{(\Gamma \dot{\gamma })}^{2}]}^{\frac{n-1}{2}},$$

where **p**, μ, I, μ_0_, μ_∞_, *Γ*, $\dot{\gamma }$ and *n* are the pressure, Identity tensor, apparent viscosity, zero shear rate viscosity, infinite shear rate viscosity, material time constant, shear rate, and power law index respectively. Here, $\frac{({\rm{\mu }}\,-{{\rm{\mu }}}_{\infty })\,}{({{\rm{\mu }}}_{0}-{{\rm{\mu }}}_{\infty })\,}$ represents the quotient in the power law region. The shear rate is given by

7 $$\dot{\gamma }=\sqrt{\frac{1}{2}\,\sum _{j}\sum _{j}{\dot{\gamma }}_{ij}{\dot{\gamma }}_{ji}}=\sqrt{\frac{1}{2}{\rm{\Pi }}}=\sqrt{\frac{1}{2}tr({{{\bf{A}}}_{1}}^{2})}.$$

Here Π represents the second invariant strain rate tensor.

8 $${{\bf{A}}}_{1}=(grad\,{\bf{V}})+{(grad{\bf{V}})}^{{\rm{T}}},$$

with **A**_1_ is the Rivlin-Erickson tensor. In most practical cases μ_0_ $\gg$ μ_∞_ and μ_∞_ is taken as zero. Utilizing Eqs (6) and (5) takes the form

9 $${\boldsymbol{\tau }}=-{\rm{p}}{\bf{I}}+{{\rm{\mu }}}_{0}{[1+{(\Gamma \dot{\gamma })}^{2}]}^{\frac{n-1}{2}}{{\bf{A}}}_{1}.$$

The range of power law index *n* varies between 0 and 1 *i.e*., 0 < *n* < 1 represents the shear thinning or pseudo plastic fluids, *n* > 1 signifies the shear thickening or dilatant fluids and *n* = 1 denotes the Newtonian fluids.

### Mathematical formulations

**Figure 1:**
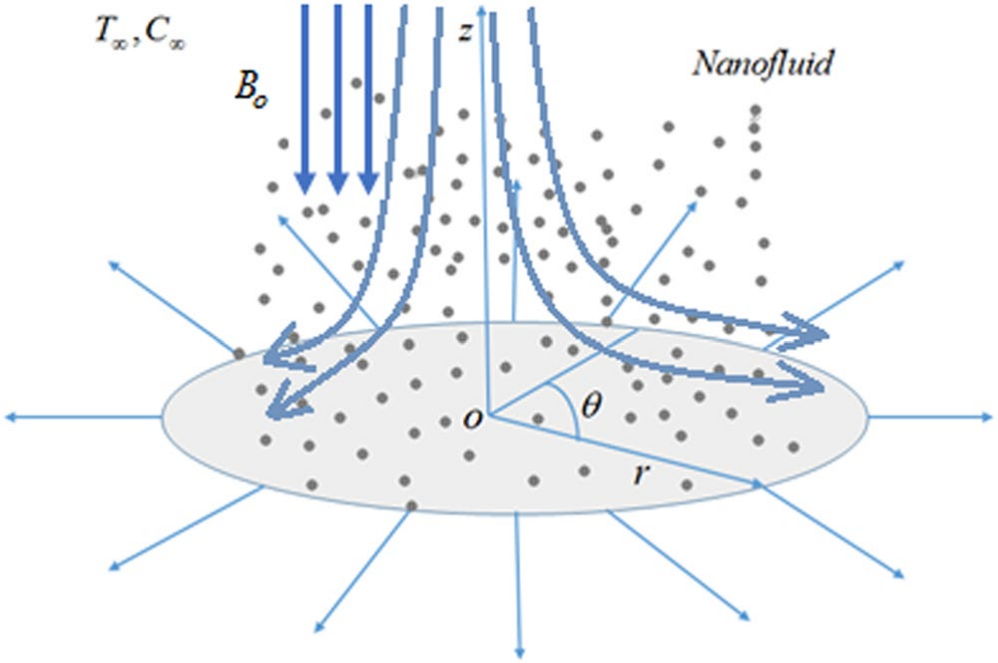
Flow geometry.

Consider the flow, heat and mass transfer of axisymmetric two-dimensional and incompressible Carreau nanofluid. A uniform magnetic field that has a strength *B*_0_ is executed in *z*-direction in the absence of an induced magnetic field. The sheet is radially stretched with stretching velocity *u*_*w*_(*r*) = *ar*, in which *r* is distance from the origin and *a* is a positive constant. The sheet that coincides with the plane *z* = 0 and fluid is bounded in *z* ≥ 0. The system of cylindrical polar coordinate (*r*, *θ*, *z*), was chosen for mathematical illustrations (see Fig. 1). Features of heat transfer mechanism is scrutinized in view of heat generation/absorption and nonlinear radiation. The mass transfer phenomenon with chemical reaction is also retained by utilizing the impressions of Brownian motion and thermophoresis. Additionally, the zero-mass flux condition at the surface is also incorporated. The uniform temperature at the surface is *T*_*w*_ and far away from the surface is *T*_∞_ such that *T*_*w*_ > *T*_∞_. At the surface of sheet, the concentration of nanoparticles is restrained by

10 $${D}_{B}\frac{{\rm{\partial }}C}{{\rm{\partial }}z}+\frac{{D}_{T}}{{T}_{{\rm{\infty }}}}\frac{{\rm{\partial }}T}{{\rm{\partial }}z}=0,$$

and far away from the surface i.e., the ambient concentration is taken to be *C*_∞_ which is constant.

For two-dimensional axisymmetric flow, velocity, concentration fields and temperature and are taken in the following fashion

11 $${\boldsymbol{V}}=[u(r,z),\,0,\,w(r,z)],\,T=T(r,z),\,\varphi =\varphi (r,\,z).$$

Now, inserting Eq. (11) into Eqs (1)–(4) keeping in the mind the results obtained in Eqs (6) and (7). Laborious and straight forward calculations end in the following set of boundary layer equations under the suppositions for the Carreau nanofluid^5^ are as below:

12 $$\frac{\partial u}{\partial r}+\frac{u}{r}+\frac{\partial w}{\partial z}=0,$$

13 $$u\frac{\partial u}{\partial r}+w\frac{\partial u}{\partial z}=\nu \frac{{\partial }^{2}u}{\partial {z}^{2}}{[1+{{\rm{\Gamma }}}^{2}{(\frac{\partial u}{\partial z})}^{2}]}^{\frac{n-1}{2}}+\nu (n-1){{\rm{\Gamma }}}^{2}\frac{{\partial }^{2}u}{\partial {z}^{2}}{(\frac{\partial u}{\partial z})}^{2}{[1+{{\rm{\Gamma }}}^{2}{(\frac{\partial u}{\partial z})}^{2}]}^{\frac{n-3}{2}}-\,\frac{\sigma {Bo}^{2}}{\rho }u,$$

14 $$u\frac{\partial T}{\partial r}+w\frac{\partial T}{\partial z}=\alpha \frac{{\partial }^{2}T}{\partial {z}^{2}}+\tau [{D}_{B}\frac{\partial C}{\partial z}\frac{\partial T}{\partial z}+\frac{{D}_{T}}{{T}_{\infty }}{(\frac{\partial T}{\partial z})}^{2}]+\frac{Q}{\rho {c}_{f}}(T-{T}_{\infty })-\frac{1}{\rho {c}_{p}}\frac{\partial {q}_{r}}{\partial z}$$

15 $$u\frac{\partial C}{\partial r}+w\frac{\partial C}{\partial z}=[{D}_{B}\frac{{\partial }^{2}C}{\partial {z}^{2}}+\frac{{D}_{T}}{{T}_{\infty }}\frac{{\partial }^{2}T}{\partial {z}^{2}}]-{K}_{r}(C-{C}_{\infty }),$$

with allied boundary conditions

16 $$\begin{array}{c}u={u}_{w}(r)=ar,\,w=0,\,T={T}_{w},\,{D}_{B}\frac{{\rm{\partial }}C}{{\rm{\partial }}z}+\frac{{D}_{T}}{{T}_{{\rm{\infty }}}}\frac{{\rm{\partial }}T}{{\rm{\partial }}z}=0;\,at\,z=0\\ \quad \quad \quad \quad \quad \,\,u\to 0,T\to {T}_{{\rm{\infty }}},C\to {C}_{{\rm{\infty }}},\,as\,z\to {\rm{\infty }},\end{array},$$

where *u* and *w* signify the velocities along *r* and *z* directions, individually, *B*_0_ is the strength of magnetic field, Γ is the material constant, *n* is a power law index, $\nu =\frac{{\mu }_{0}}{\rho }$ is the kinematic viscosity such that *μ*_0_ stands for dynamic viscosity and *ρ* denotes the fluid’s density, $\tau =\frac{{(\rho c)}_{p}}{{(\rho c)}_{f}}$ is the quotient of heat capacity of the nanoparticles to the heat capacity of the primary fluid, $\alpha =\frac{k}{\rho {c}_{p}}$ is the thermal diffusivity such that *k* depicts the thermal conductivity and *c*_*p*_ portrays the specific heat, *T* is the temperature distribution, *C* is the nanoparticles volume fraction, *D*_*B*_ and *D*_*T*_ are the “Brownian motion and thermophoresis” diffusion coefficients, individually. Moreover, *Q* signifies the heat generation/absorption coefficient, *K*_*r*_ is the rate of chemical reaction and *q*_*r*_ signify the Rosseland’s radiation flux. Here, the mass flux is taken to be zero to get rid of undesirable effects and to get maximum heat transfer output^43^.

In the view of Rosseland approximation, the reduced form of radiation flux can be written as:

17 $${q}_{r}=-\frac{4{\sigma }^{\ast }}{3{k}^{\ast }}\frac{\partial {T}^{4}}{\partial z}.$$

Here, σ^*^and k^*^are Stefan-Boltzmann constant and Mean absorption coefficient. We consider the temperature of nanoparticles is so small so that *T*^4^ can be expanded about the free stream temperature *T*_0_ as follows:

18 $${\check{T}}^{4}={{\check{T}}_{0}}^{4}+4{{\check{T}}_{0}}^{3}({\check{T}}-{\check{T}}_{0})+6{{\check{T}}_{0}}^{2}{({\check{T}}-{\check{T}}_{0})}^{2}+\ldots $$

Ignoring the higher order terms, we left with

19 $${\check{T}}^{4}\cong {{\check{T}}_{0}}^{4}+4{{\check{T}}_{0}}^{3}({\check{T}}-{\check{T}}_{0})$$

Utilizing Eq. (20) into Eq. (18), we come to

20 $${q}_{r}=-\frac{16{\sigma }^{\ast }}{3{k}^{\ast }}{T}^{3}\,\frac{{\rm{\partial }}T}{{\rm{\partial }}z}.$$

It is stated that contrary to traditional cases we have assumed the nonlinear structure of thermal radiation.

We set up the similarity transformations here as under:

21 $$\eta =z\sqrt{\frac{a}{\nu }},\,{\rm{\Psi }}(r,\,z)=-{r}^{2}\sqrt{a\nu }f(\eta ),\,\theta (\eta )=\frac{T-{T}_{\infty }}{{T}_{w}-{T}_{\infty }},\,\varphi (\eta )=\frac{C-{C}_{\infty }}{{C}_{\infty }},$$

where Ψ(z, r) is the stream function having the following property

22 $${u}_{r}=-\frac{1}{r}\frac{{\rm{\partial }}{\rm{\Psi }}}{{\rm{\partial }}z},\,{w}_{z}=\frac{1}{r}\frac{{\rm{\partial }}{\rm{\Psi }}}{{\rm{\partial }}r}.$$

In Eq. (21), *θ* and *ϕ* are dimensionless temperature and nanoparticles concentration respectively and *η* is the dimensionless independent variable. With the help of Eqs (21) and (22), requirement of equation of continuity is automatically fulfilled while Eqs (13–16), take the form:

23 $$\{1+nW{e}^{2}{(f^{\prime\prime} )}^{2}\}{\{1+W{e}^{2}{(f^{\prime\prime} )}^{2}\}}^{\frac{n-3}{2}}\,f\prime\prime\prime +\,2ff^{\prime\prime} -\,{f^{\prime} }^{2}-{M}^{2}f^{\prime} =0,$$

24 $$\theta ^{\prime\prime} +2Prf\theta ^{\prime} +Pr{N}_{b}\theta ^{\prime} \varphi ^{\prime} +Pr{N}_{t}\theta {\text{'}}^{2}+Pr\delta \theta +\frac{4}{{N}_{R}}[\frac{1}{3}{\{1+({\theta }_{w}-1)\theta \}}^{3}\theta ^{\prime\prime} +{\{1+({\theta }_{w}-1)\theta \}}^{2}({\theta }_{w}-1)\theta {\text{'}}^{2}]\,=0,$$

25 $$\varphi ^{\prime\prime} +2Scf\varphi ^{\prime} -Sc\gamma \varphi +\frac{{N}_{t}}{{N}_{b}}\theta \text{'}\text{'}=0,$$

subject to the transformed boundary conditions

26 $$f(0)=0,\,f^{\prime} (0)=1,\,{\rm{\theta }}(0)=1,\,{N}_{b}\varphi ^{\prime} (0)+{N}_{t}\theta ^{\prime} (0)=0,\,f^{\prime} ({\eta }_{\infty })\to 0,\,{\rm{\theta }}({\eta }_{\infty })\to 0,\,\,\varphi ({\eta }_{\infty })\to 0.$$

In the aforementioned equations, primes denote differentiation with respect to *η*.δ < 0 refers to heat generation and δ < 0 represents heat absorption parameters respectively. *N*_*R*_, *N*_*b*_, *We*, *M*, *Pr*, *γ*, *θ*_*w*_, *Sc* and *N*_*t*_ denote Nonlinear radiation parameter, Brownian motion parameter, Weissenberg number, Magnetic parameter, Prandtl number, chemical reaction parameter, Schmidt number and thermophoresis parameter respectively. The dimensionless parameters are defined as:

27 $$\begin{array}{c}{We}=\frac{{{\rm{\Gamma }}}^{2}{a}^{3}{r}^{2}}{\nu },{M}^{2}=\frac{\sigma {{B}_{0}}^{2}}{\rho a},Pr=\frac{\nu \rho {c}_{p}}{k},{N}_{b}=\frac{{(\rho c)}_{p}{D}_{B}{C}_{\infty }}{{(\rho c)}_{f}\nu },\\ {N}_{t}=\frac{{(\rho c)}_{p}{D}_{T}({T}_{w}-{T}_{\infty })}{{(\rho c)}_{f}\nu {T}_{\infty }},\delta =\frac{Q}{a{(\rho c)}_{f}},{N}_{R}=\frac{k{k}^{\ast }}{4{\sigma }^{\ast }{{T}_{\infty }}^{3}},{\theta }_{w}=\frac{{T}_{w}}{{T}_{\infty }},Sc=\frac{\nu }{{D}_{B}},\gamma =\frac{{K}_{r}}{a}.\end{array}$$

### Skin friction coefficient and local Nusselt number

Two essential quantities of physical fascination are Skin friction coefficient *C*_*f*_ and local Nusselt number *Nu*_*r*_. They are characterized as follows:

28 $${C}_{f}=\frac{{\tau }_{w}{|}_{z=0}}{\rho {u}_{w}^{2}}\,N{u}_{r}=\frac{r{q}_{w}{|}_{z=0}}{k({T}_{w}-{T}_{\infty })}.$$

In Eq. (28), *τ*_*w*_ is the shear stress at the surface in the *z* − direction, surface heat flux is denoted by *q*_*w*_ and defined as under:

29 $${\tau }_{w}={\mu }_{0}\frac{\partial u}{\partial z}{[1+{{\rm{\Gamma }}}^{2}{(\frac{\partial u}{\partial z})}^{2}]}^{\frac{n-1}{2}},\,{q}_{w}=-k{(\frac{\partial T}{\partial z})}_{z=0}+{q}_{r}{|}_{z=0}.$$

Using Eqs (21), (22) and (29) in Eq. (28), we get the reduced form of skin friction coefficient and local Nusselt number as:

30 $$R{e}^{\frac{1}{2}}{C}_{f}=f^{\prime\prime} (0){\{1+W{e}^{2}{(f^{\prime\prime} )}^{2}\}}^{\frac{n-1}{2}},$$

31 $$R{e}^{-\frac{1}{2}}N{u}_{r}=-\theta ^{\prime} (0)[1+\frac{4}{3{N}_{R}}{\{1+({\theta }_{w}-1)\theta (0)\}}^{3}],$$

where $Re=\frac{r{u}_{w}}{\nu }$ specify the local Reynolds number.

## Numerical Procedure

The nonlinear partially coupled ordinary differential Eqs (23–25) along with boundary conditions (26) are integrated with the aid of MATLAB tool bvp4c^44^. To attain this, the partially coupled ordinary differential equations are first renovated to first order ordinary differential equations. Using the succeeding substitutions

32 $$f={y}_{1},f^{\prime} ={y}_{2},f^{\prime\prime} ={y}_{3},\,\theta ={y}_{4},\,\theta ^{\prime} ={y}_{5},\varphi ={y}_{6},\,\varphi ^{\prime} ={y}_{7},$$

33 $${y}_{1}^{\prime} ={y}_{2},{y}_{2}^{\prime} ={y}_{3},\,{y}_{3}^{\prime} =\frac{-2{y}_{1}{y}_{3}+{{y}_{2}}^{2}+M{y}_{2}}{\{1+nW{e}^{2}{{y}_{3}}^{2}\}{\{1+W{e}^{2}{{y}_{3}}^{2}\}}^{\frac{n-3}{2}}},$$

34 $${y}_{4}^{\prime} ={y}_{5},{y}_{5}^{\prime} =-\frac{2Pr{y}_{1}{y}_{5}+Pr{N}_{b}{y}_{5}{y}_{7}+Pr{N}_{t}{{y}_{5}}^{2}+Pr\delta {y}_{4}+\frac{4}{{N}_{R}}{\{1+({\theta }_{w}-1){y}_{4}\}}^{2}({\theta }_{w}-1){{y}_{5}}^{2}}{1+\frac{4}{3{N}_{R}}{\{1+({\theta }_{w}-1){y}_{4}\}}^{3}},$$

35 $${y}_{6}^{\prime} ={y}_{7},\,{y}_{6}^{\prime} =-2Sc{y}_{1}{y}_{7}+Sc\gamma {y}_{6}-\,\frac{{N}_{t}}{{N}_{b}}{y}_{5}^{\prime} .$$

The boundary conditions take the following structure

36 $${y}_{1}(0)=0,{y}_{2}(0)-1=0,{y}_{4}(0)-1=0,{N}_{b}{y}_{7}(0)+{N}_{t}{y}_{5}=0,\,{y}_{2}({\eta }_{\infty })\to 0,{y}_{4}({\eta }_{\infty })\to 0,{y}_{6}({\eta }_{\infty })\to 0.$$

The MATLAB tool bvp4c incorporates basically the finite difference code as a default. This method is typically a collocation method of order-four. Here, the mesh choice and error mechanism are fortified by the residual of continuous solution. The tolerance was fixed to 10^−7^.In this method, the decision of *η*_*∞*_ = 10, guarantees that every numerical solution approach asymptotic values accurately.

## Results and Discussion

Numerical solutions of Eqs (23–25)supported with the boundary conditions (26) have been calculated utilizing a MATLAB built-in tool bvp4c. Computations have been done for distinct values of all arising important parameters. The impacts of these parameters on temperature, velocity and nanoparticle concentration profiles are inspected with the assistance of graphical data. The admissible ranges of involved physical parameters^45^ are 0.5 ≤ *M* ≤ 2.0, 0.05 ≤ *We* ≤ 6.0, 2.5 ≤ *Pr* ≤ 4.0, 0.03 ≤ *δ* ≤ 0.35, 1.0 ≤ *N*_*R*_ ≤ 2.2, 1.1 ≤, *θ*_*w*_ ≤ 1.7, 0.5 ≤ *N*_*t*_ ≤ 3.5, 0.1 ≤ *γ* ≤ 0.7, 0.3 ≤ *N*_*b*_ ≤ 0.9, 1.0 ≤ *Sc* ≤ 2.5. Also, numerical results of local Nusselt number and skin friction coefficient are tabulated for more robust comprehension of the computed results. Furthermore, we have taken *M* = *n* = *N*_*b*_ = *N*_*t*_ = 0.5, *We* = 0.05, *Pr* = 2.5, *N*_*R*_ = *Sc* = 1.0, *θ*_*w*_ = 1.1, *δ* = 0.03 and *γ* = 0.1 fix, by varying these parameters one by one throughout the graphical illustrations.

For the verification of current numerical routine, a comparison is performed with the existing articles in a limiting case. Our computed results of skin friction coefficient for several values of magnetic parameter *M* is compared with those of Makinde *et al*.^31^. and Azam *et al*.^46^. (see Table 1). It is noted that our processed results show better outstanding with those of Makinde and Azam. Thus, trustworthy results are being presented here.

**Table 1:** A comparison of values of *f*″(0) for varied values of *M* when *We* = 0 and *n* = 1.

*M* ^2^	Makinde *et al*.^31^	Azam *et al*.^46^	Present results
0.0	−1.17372	−1.17372	−1.173720
0.5	−1.36581	−1.36581	−1.365814
1.0	−1.53571	−1.53571	−1.535709
2.0	−1.83049	−1.83049	−1.830490
3.0	−2.08484	−2.08485	−2.084846

The upshots of *M*, *We*, *n*, *N*_*R*_, *θ*_*w*_, *δ* and *N*_*t*_ on Skin friction coefficient and local Nusselt number are enumerated through Tables 2 and 3. The data in these tables suggest that the amount of skin friction coefficient intensifies as we escalate the values of magnetic parameter and power law index, while the inverse pattern is noted for Weissenberg number. From these tables, it can also be seen that the local Nusselt number is a decrementing function of magnetic parameter and Weissenberg number, while the impression of power law index is to depreciate the magnitude of local Nusselt number. Furthermore, the local Nusselt number declines by the incremented values of nonlinear radiation parameter, heat absorption parameter and thermophoresis parameter, while the reverse progression is noted for the temperature ratio parameter.

**Table 2:** Numerically calculated values of skin friction $-R{e}^{\frac{1}{2}}{C}_{f}$ for varied values of *M*,*We* and *n*.

*M*	*We*	*n*	$-R{e}^{\frac{1}{2}}{C}_{f}$
0.5			1.3653398
1.0			1.5350602
1.5			1.6884948
2.0			1.8294374
	0.05		1.3653398
	2.0		1.0492851
	4.0		0.85467461
	6.0		0.75112133
		0.5	1.3653398
		1.0	1.3658144
		1.5	1.3662882
		2.0	1.3667611

**Table 3:** Numerically calculated values of Nusselt number $R{e}^{\frac{-1}{2}}N{u}_{r}$ for varied values of *M*, *We*, *n*, *N*_*R*_, *θ*_*w*_, *δ* and *N*_*t*_ when *Pr* = 2.5 is fixed.

*M*	*We*	*n*	*N*_*R*_	*θ*_*w*_	*δ*	*N*_*t*_	$R{e}^{\frac{-1}{2}}N{u}_{r}$
0.5							1.8735119
1.0							1.7842637
1.5							1.7054868
2.0							1.6351292
	0.05						1.8735119
	2.0						1.6490202
	4.0						1.4700974
	6.0						1.3552638
		0.5					1.8735119
		1.0					1.8738067
		1.5					1.874101
		2.0					1.8743946
			1.0				1.8735119
			1.4				1.366576
			1.8				1.0713273
			2.2				0.87969914
				1.1			1.8735119
				1.3			2.0211788
				1.5			2.1671585
				1.7			2.3030379
					0.03		1.8735119
					0.15		1.6188128
					0.25		1.3606069
					0.35		1.0163158
						0.5	1.8735119
						1.5	1.5985311
						2.5	1.3508399
						3.5	1.1362272

**Figure 2:**
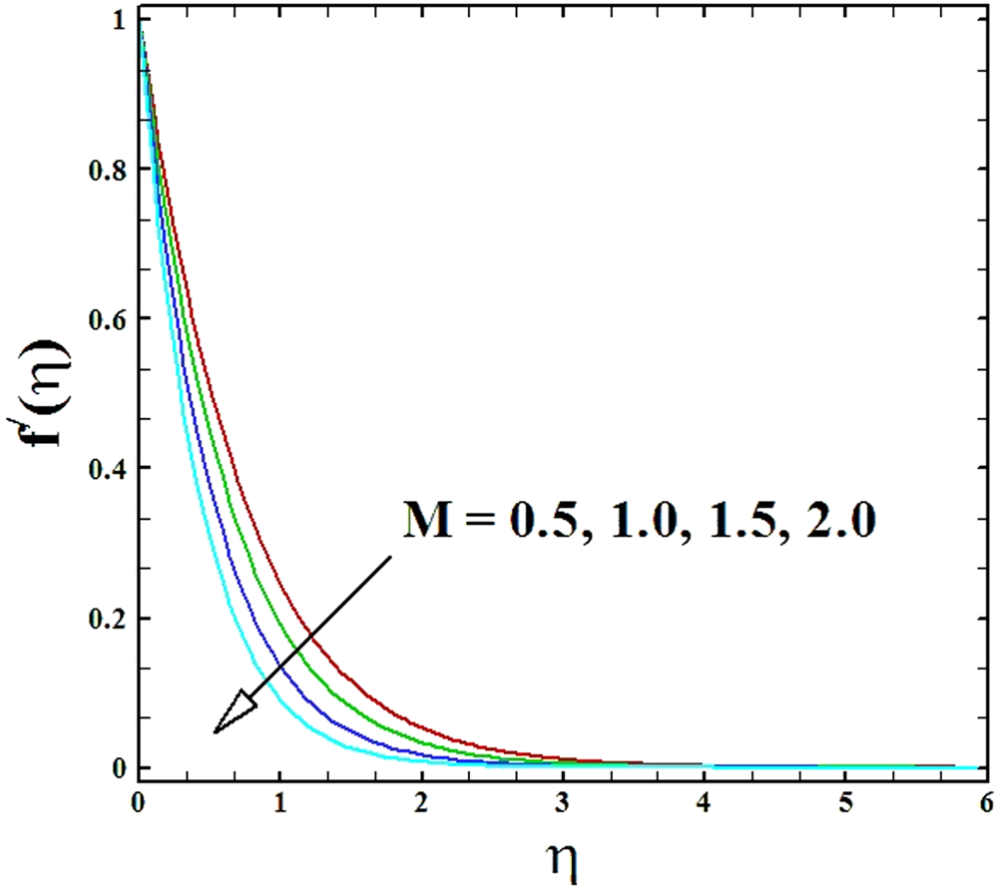
Graph of **M** versus ***f′*** (***η***).

**Figure 3:**
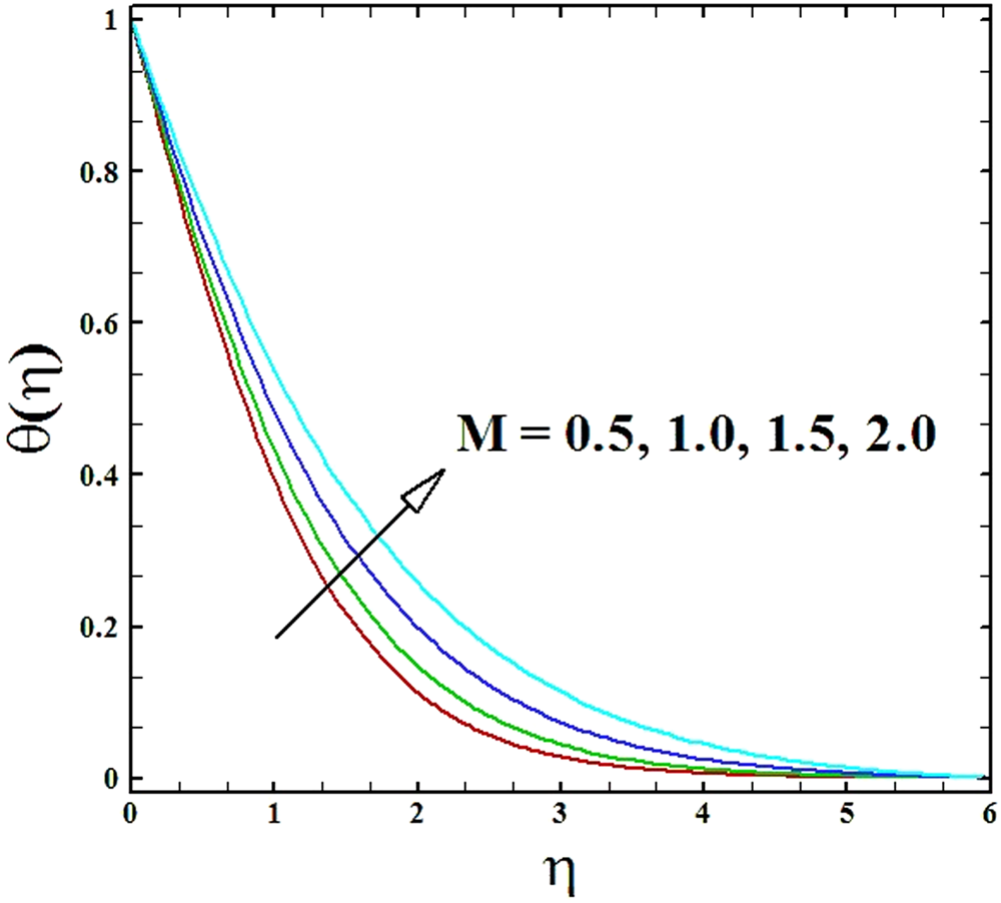
Graph of **M** versus *θ*(*η*).

**Figure 4:**
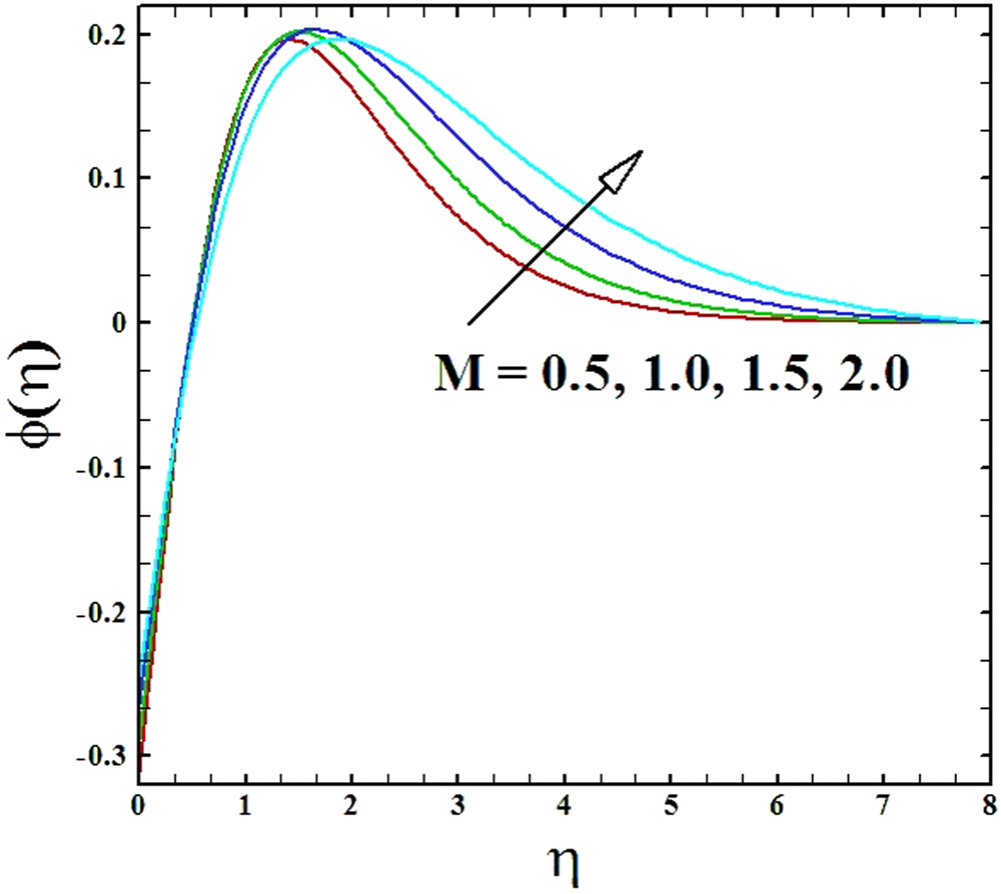
Graph of M versus ***ϕ***(***η***).

The upshots of magnetic parameter *M* on velocity, temperature and nanoparticles concentration distributions are inspected in Figs (2–4). It is perceived that as parameter *M* is incremented, the fluid velocity *f*'(*η*) diminishes. Moreover, the momentum boundary layer gets thinner as magnetic parameter value is raised, while the comportment of temperature *θ*(*η*) and nanoparticles concentration *ϕ*(*η*) enhances with the increment in magnetic parameter.

**Figure 5:**
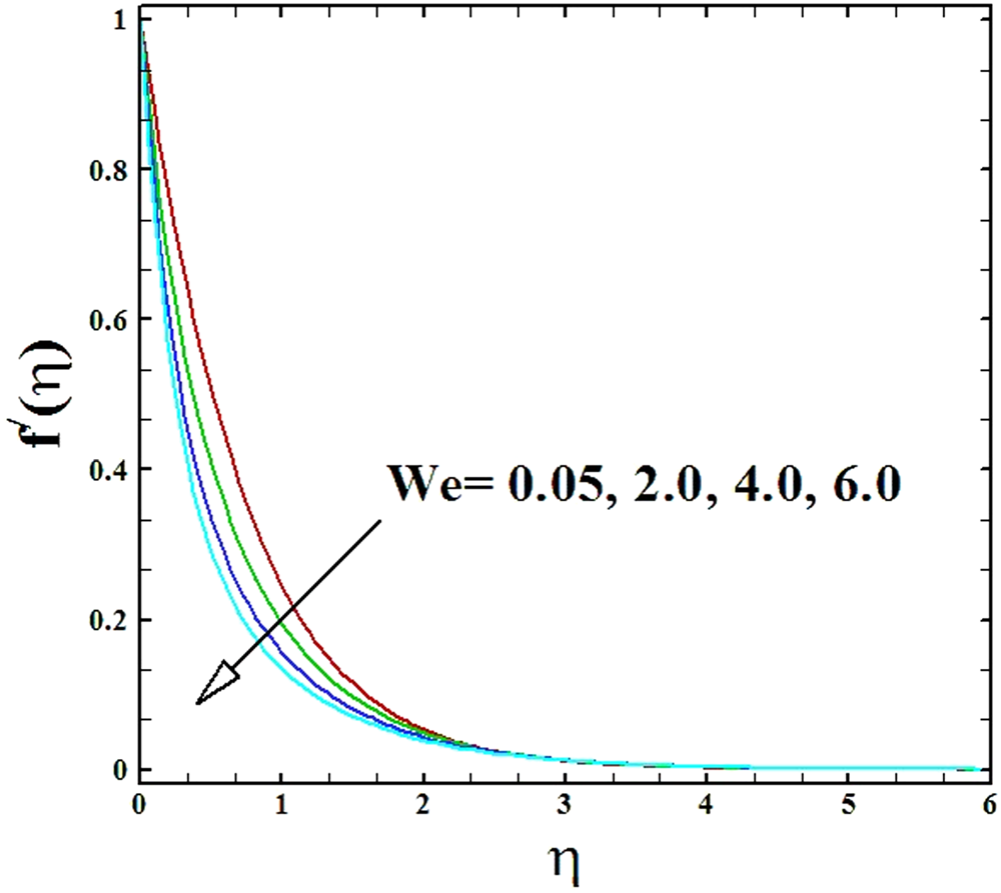
Graph of We versus ***f′***(***η***).

**Figure 6:**
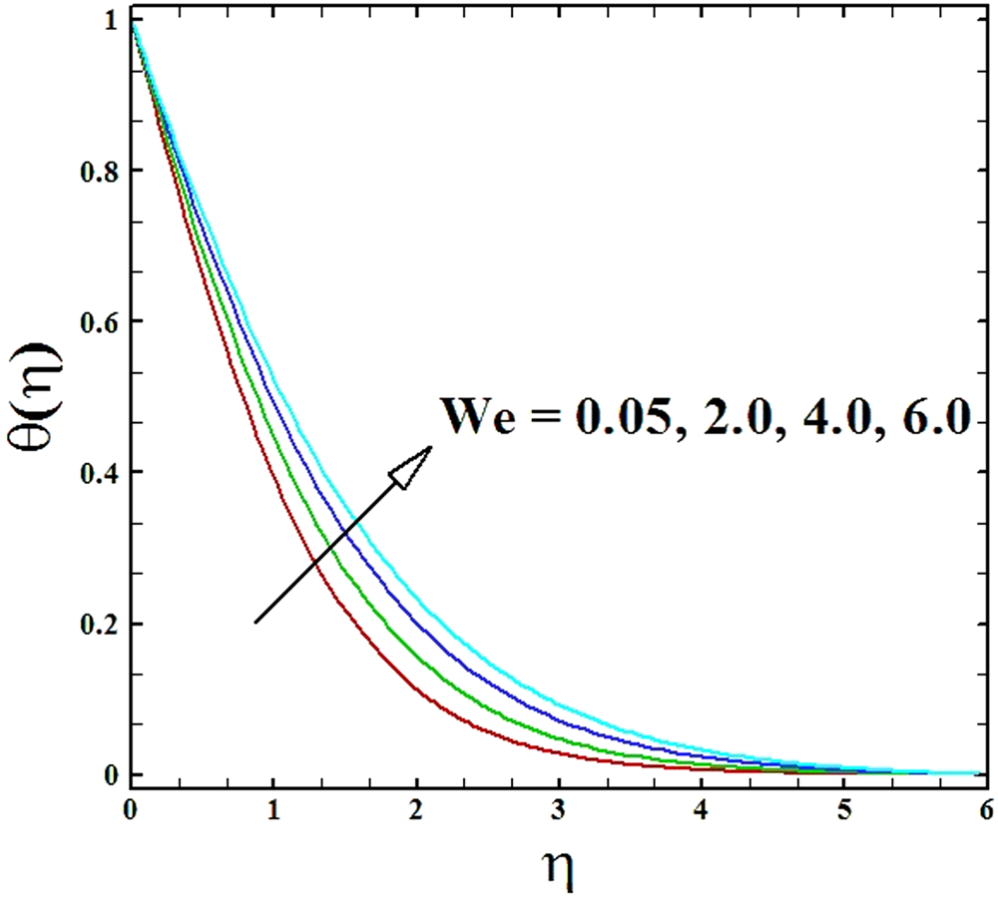
Graph of We versus ***θ***(***η***).

**Figure 7:**
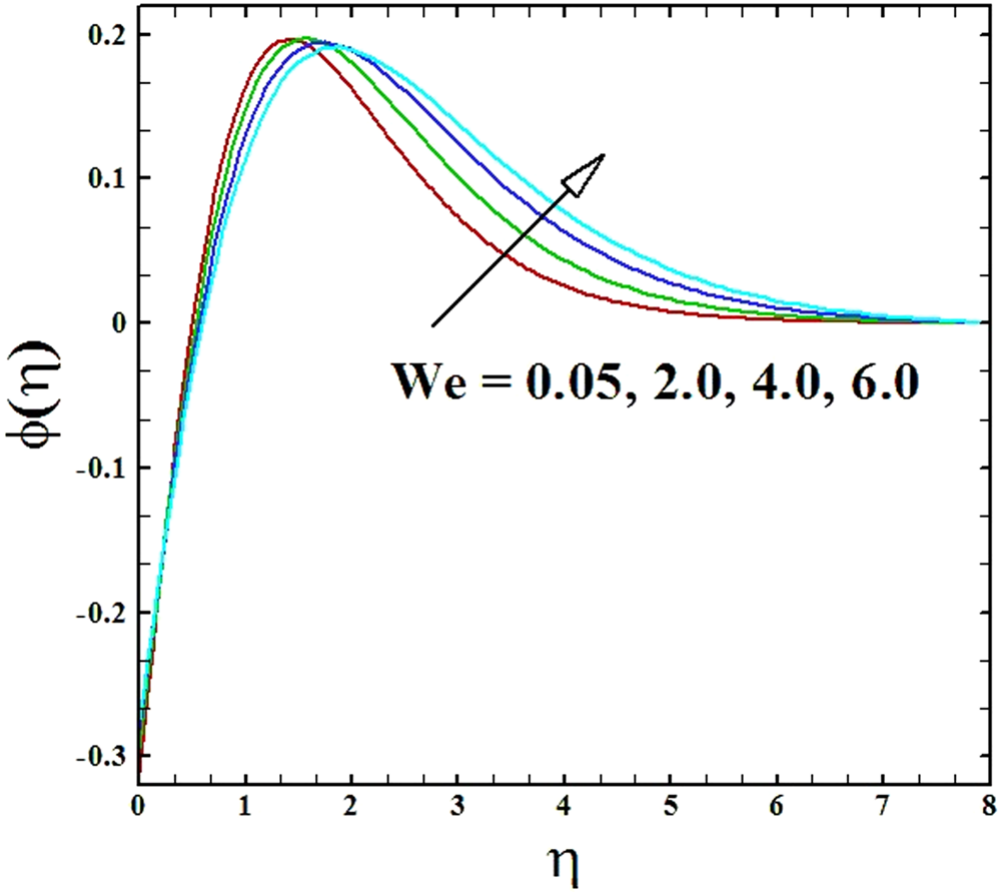
Graph of **We** versus ***ϕ***(***η***).

The upshot of Wiesenberg number *We* on the velocity, temperature and nanoparticles concentration fields has been explored and displayed in Figs (5–7). It is obvious from these illustrations that the velocity distribution declines with rise in values of Wiesenberg parameter *We*, while the manner of heat and mass transfer are found to be depress by the uplifting values of *We*.

**Figure 8:**
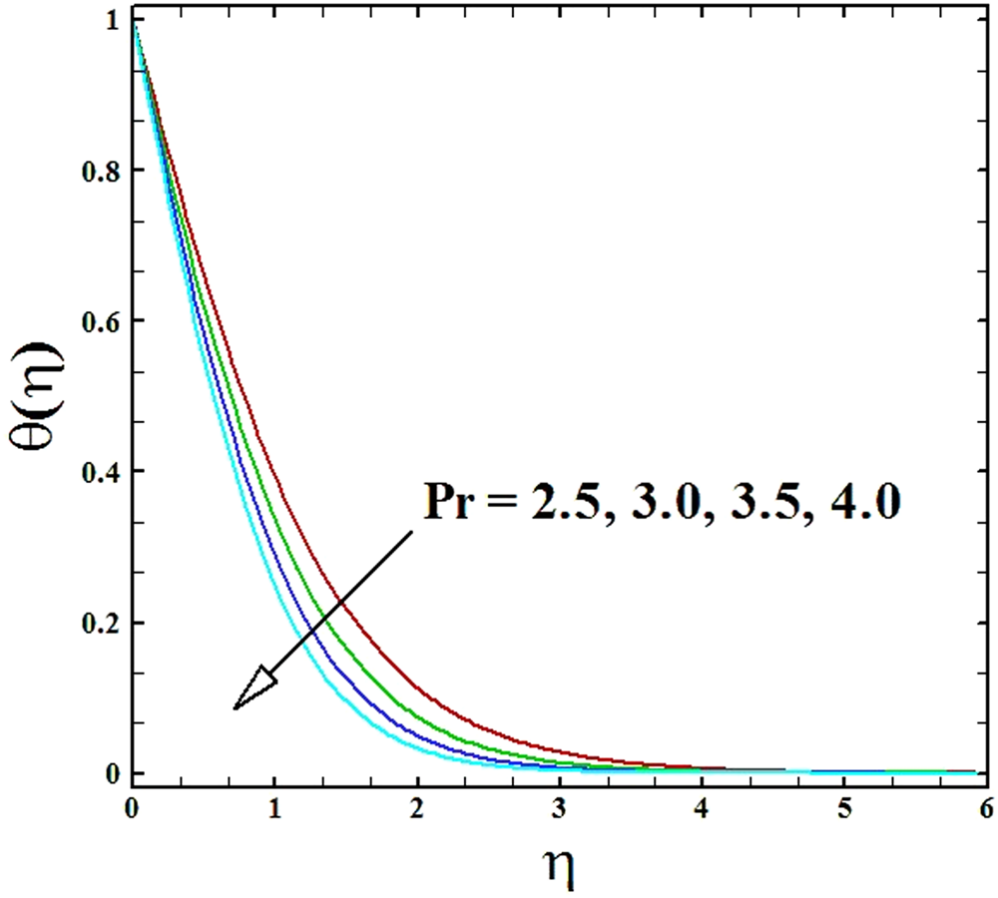
Graph of **Pr** versus *θ*(*η*).

**Figure 9:**
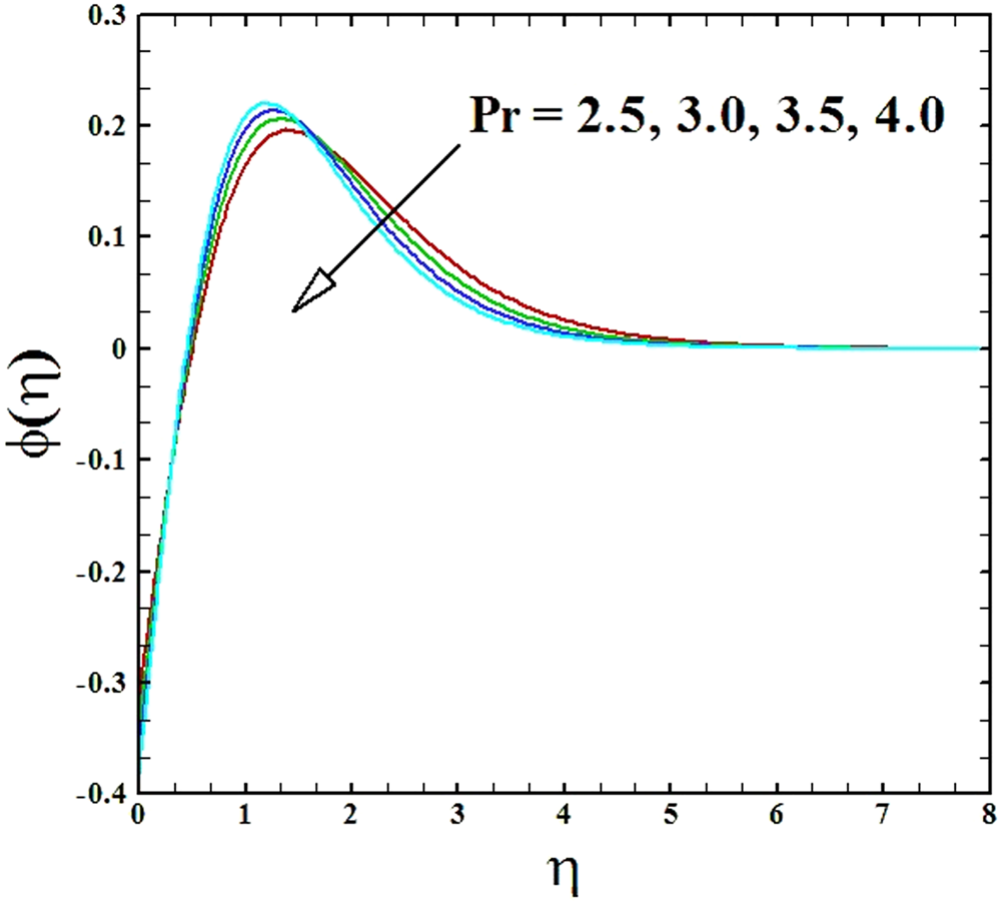
Graph of **Pr** versus *ϕ*(*η*).

The impact of Prandtl number *Pr* on temperature and nanoparticles concentration distributions are drafted in Figs (8 and 9). It can be perceived effortlessly that for the rise in Prandtl number, both the temperature and nanoparticles concentration distributions decrease. The reduction in temperature profile occurs in the way that high Prandtl number implies low thermal conductivity, as a result the fluid accomplishes lower temperature at high Prandtl number.

**Figure 10:**
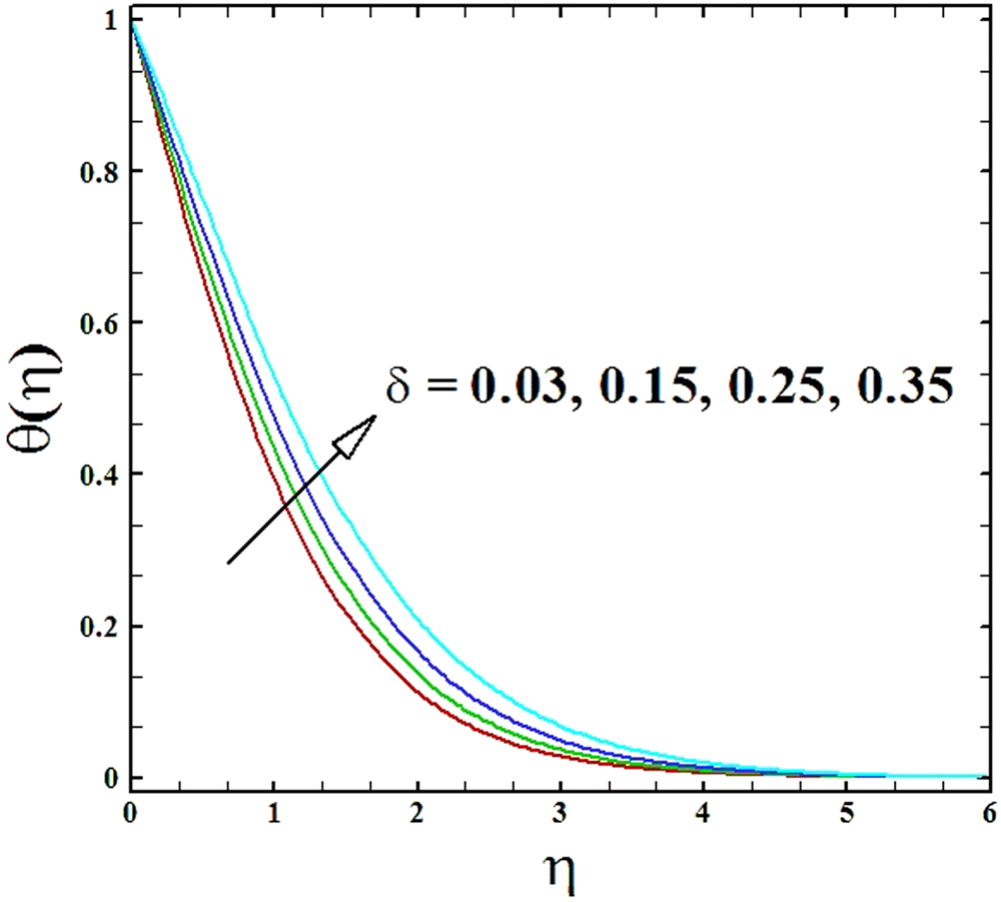
Graph of *δ* versus *θ*(*η*).

**Figure 11:**
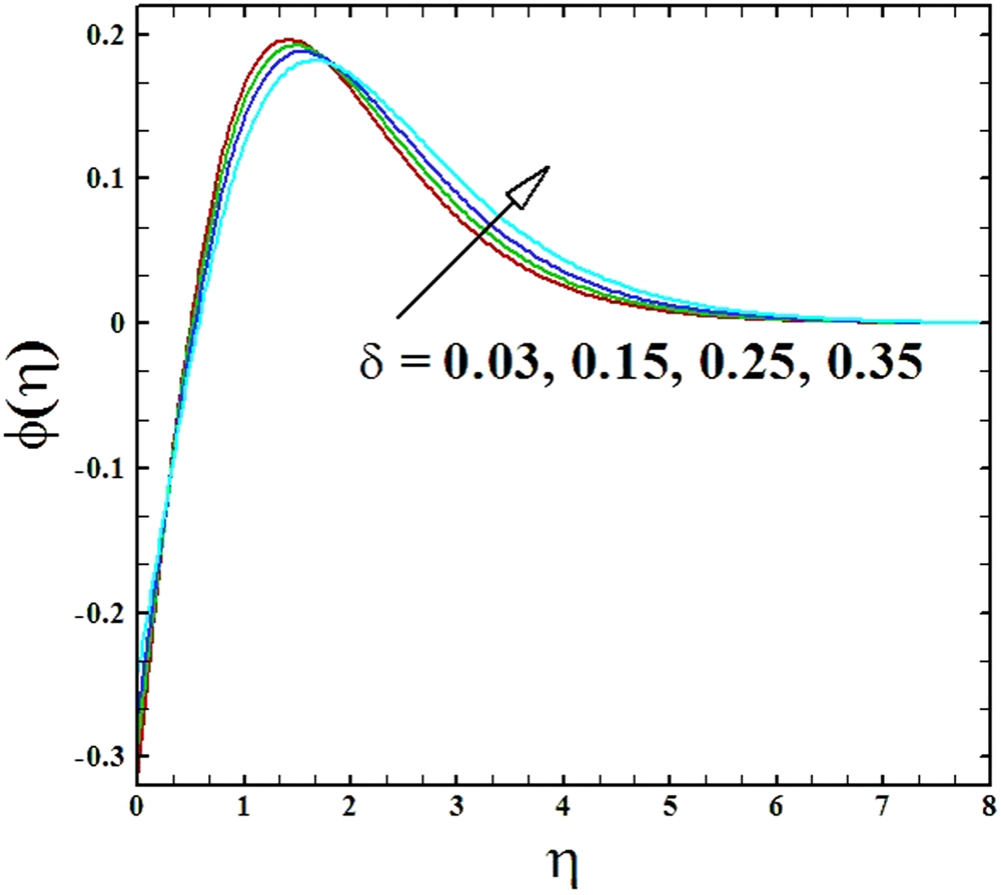
Graph of *δ* versus *ϕ*(*η*).

The contrasts of heat generation parameter *δ* on temperature and nanoparticles concentration profiles are established in Figs (10 and 11). It is witnessed that with the increment in heat generation parameter the temperature profile *θ*(*η*) as well as the nanoparticles concentration profile *ϕ*(*η*) increase.

**Figure 12:**
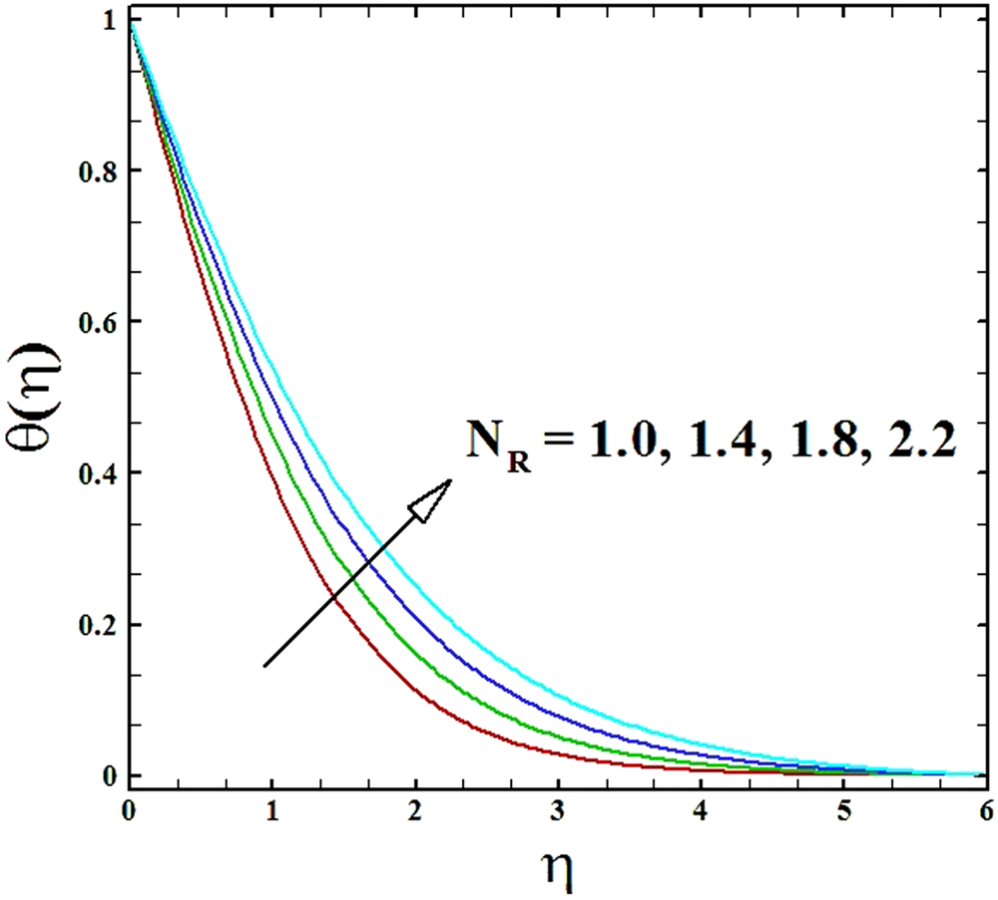
Graph of **N**_**R**_ versus *θ*(*η*).

**Figure 13:**
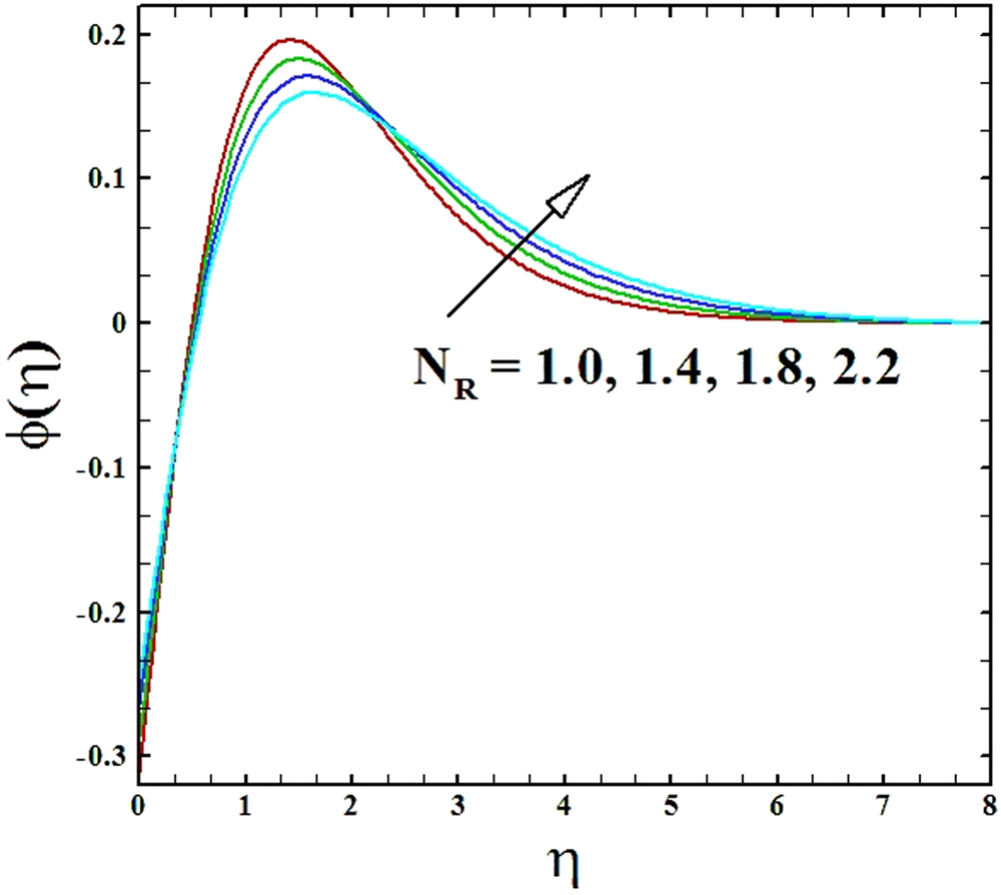
Graph of **N**_**R**_ versus *ϕ*(*η*).

Figures (12 and 13) depicts the impression of nonlinear radiation parameter *N*_*R*_ on temperature and nanoparticles concentration profiles. On evidence of these figures, it is examined that temperature and nanoparticles concentration profiles inflate by raising the values of parameter *N*_*R*_. Furthermore, the thermal boundary layer thickness boosts up strongly by escalating values of nonlinear radiation parameter.

**Figure 14:**
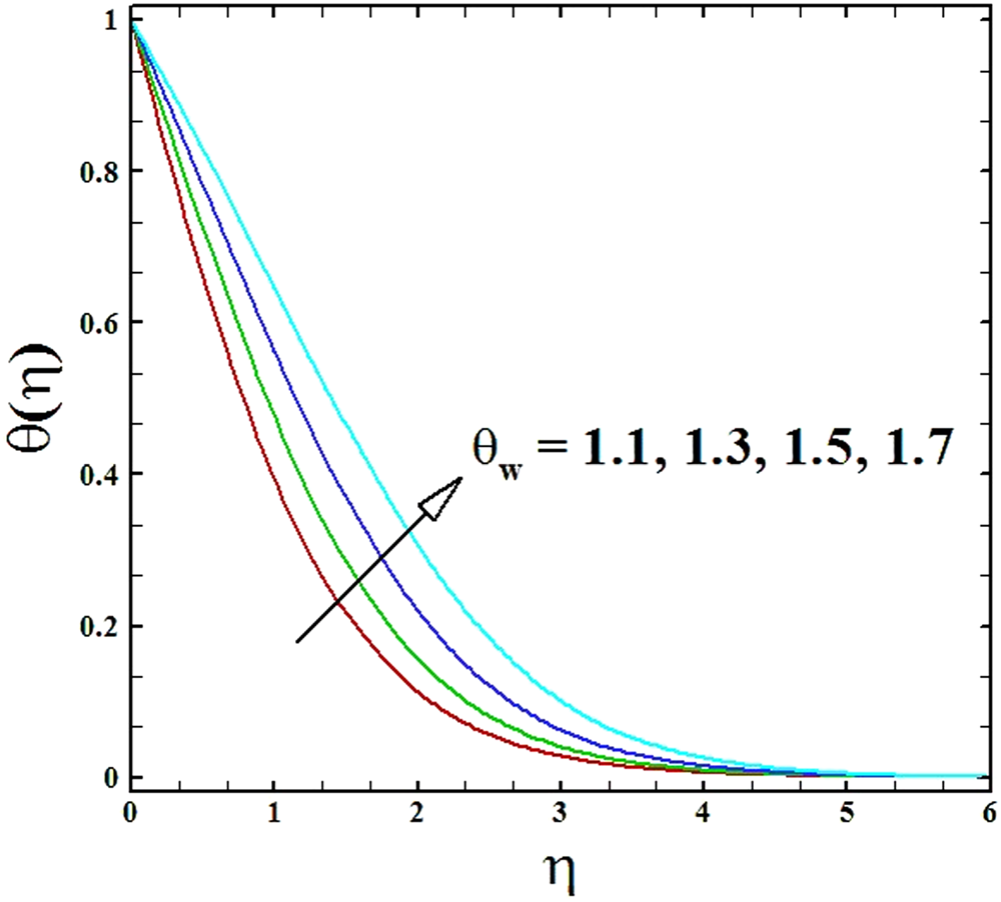
Graph of *θ*_*w*_ versus *θ*(*η*).

**Figure 15:**
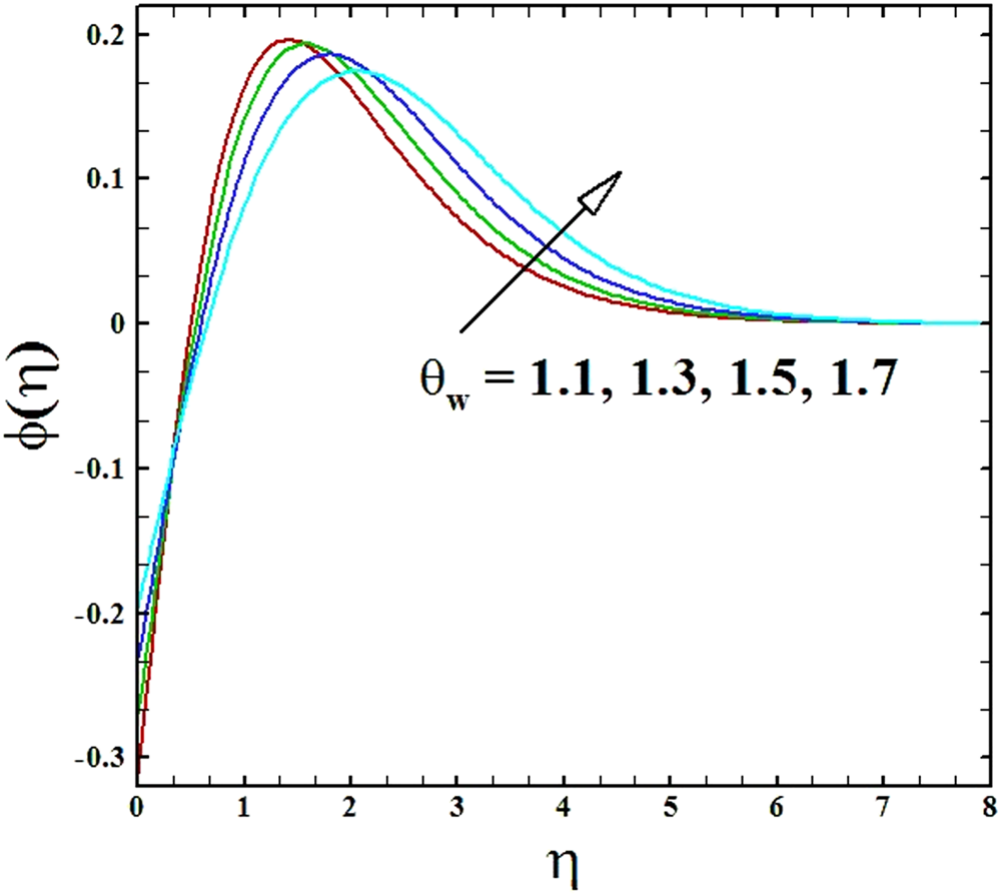
Graph of *θ*_*w*_ versus *ϕ*(*η*).

The temperature and nanoparticles concentration distributions for numerous data of temperature ratio parameter *θ*_*w*_ is organized in Figs (14 and 15). It is perceived that for mounting r values of the parameter *θ*_*w*_, the temperature and nanoparticles concentration profiles inflate. The rise in temperature profile is seen because of that increasing values of parameter *θ*_*w*_ that eventually results in elevated wall temperature in comparison to ambient temperature, subsequently fluid temperature enriches.

**Figure 16:**
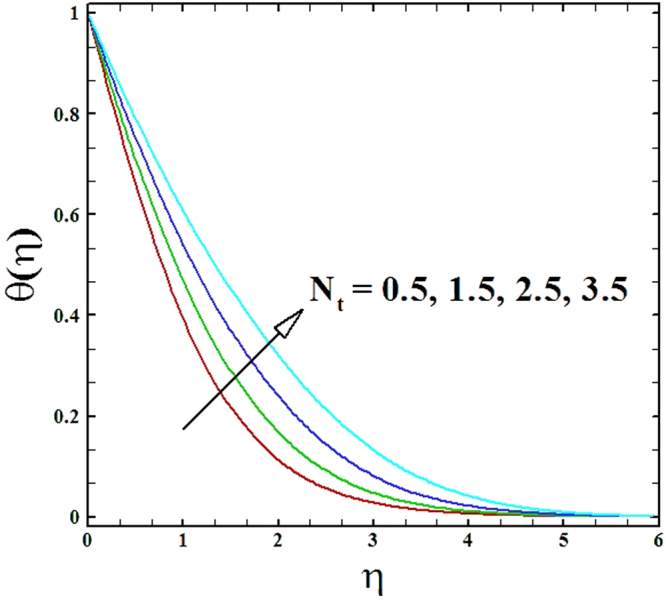
Graph of *N*_*t*_ versus *θ*(*η*).

**Figure 17:**
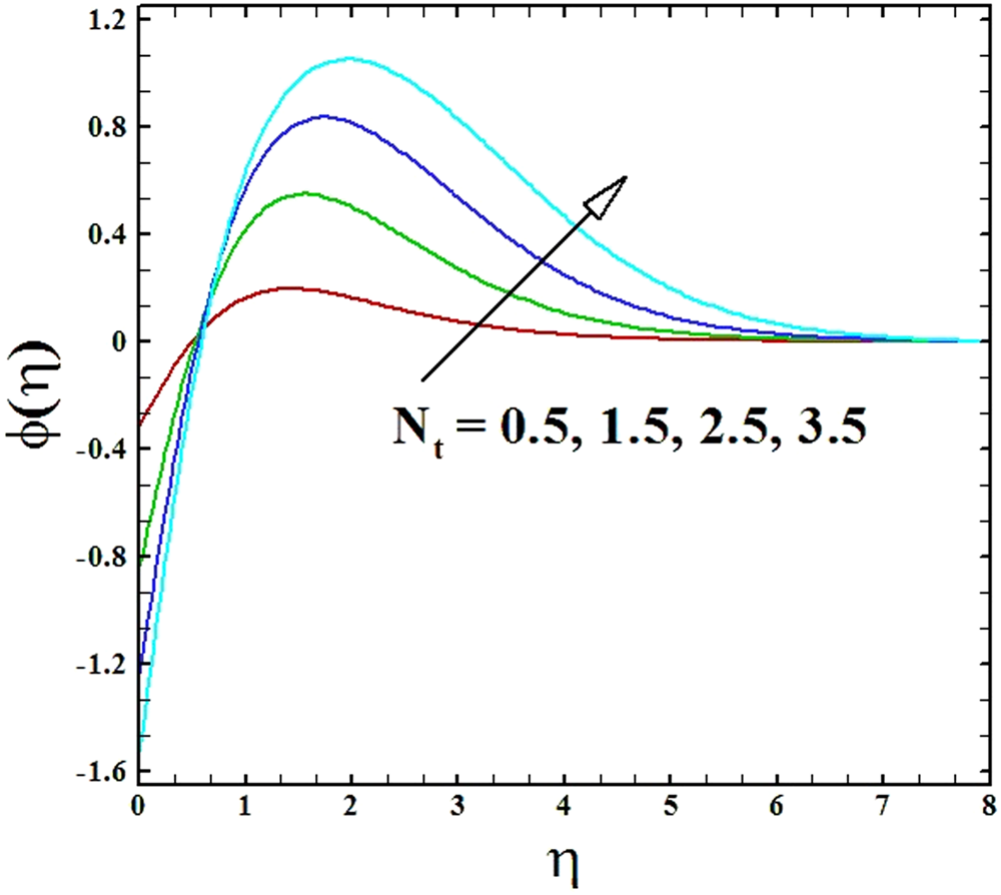
Graph of *N*_*t*_ versus *ϕ*(*η*).

The upshots of thermophoresis parameter *N*_*t*_ on temperature profiles and nanoparticles concentration profiles are portrayed in Figs (16 and 17). By these figures, it is claimed that growing values of parameter *N*_*t*_ upsurge the nanoparticles concentration and temperature profiles.

**Figure 18:**
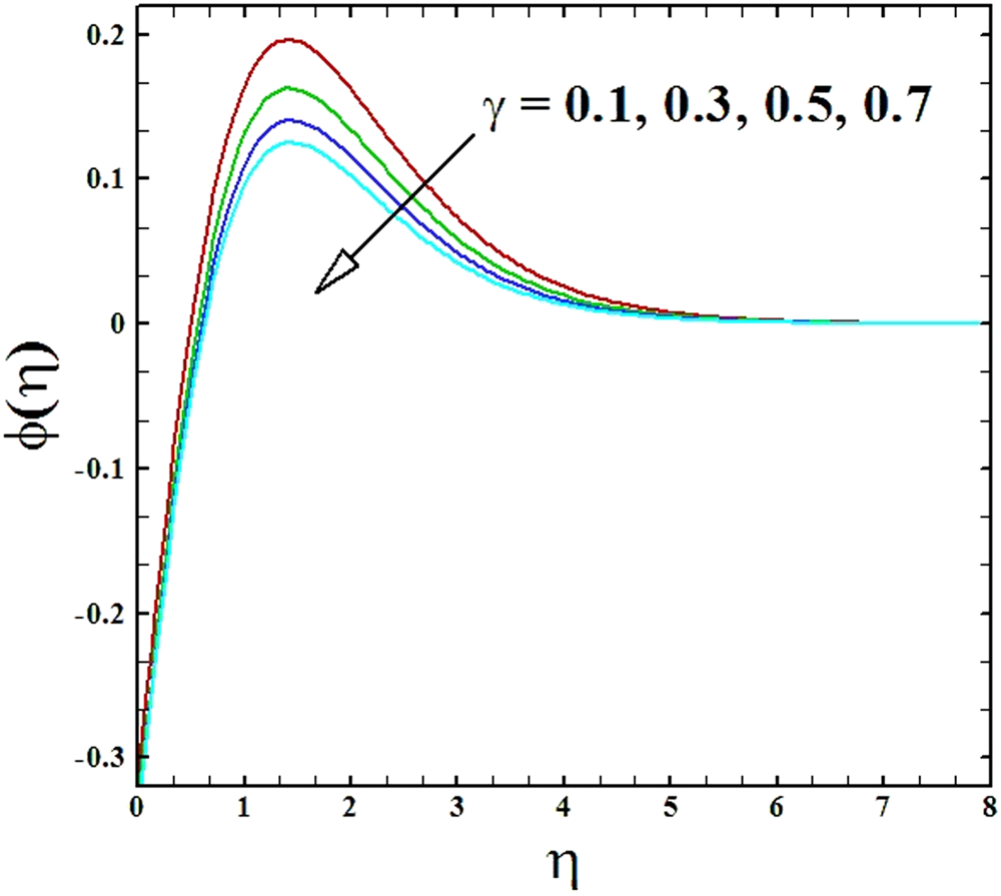
Behavior of ***ϕ***(***η***) versus escalating values of chemical reaction parameter.

To examine the effect of the chemical reaction parameter *γ* on nanoparticles concentration profile Fig. 18 is sketched. It is witnessed that as values of the parameter *γ* are raised, the nanoparticles concentration profile depresses. This occurs because of incremented values parameter *γ* which results in rise in the rate of chemical reaction and consequently the nanoparticles concentration profile reduces.

**Figure 19:**
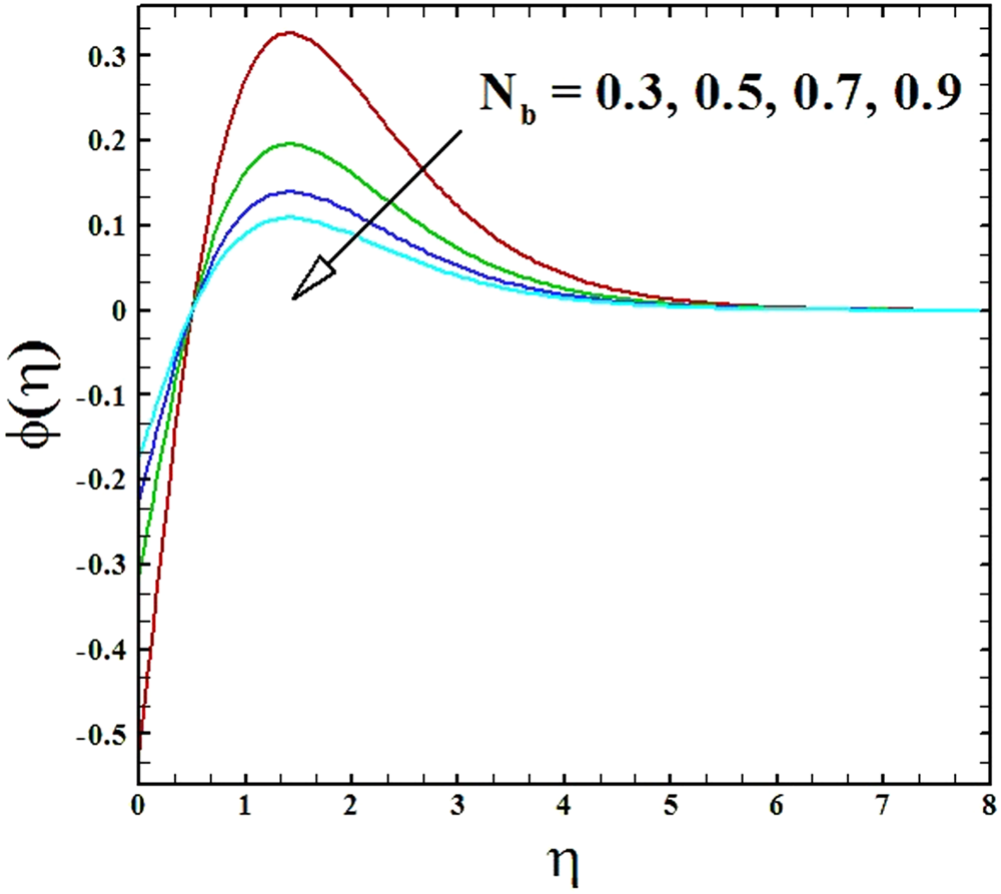
Behavior of ***ϕ***(***η***) versus escalating values of Brownian motion parameter.

The discrepancy of Brownian motion parameter *N*_*b*_ on nanoparticles concentration profile is described in Fig. 19. From this figure, it is asserted that an enrichment in the parameter *N*_*b*_ diminishes the nanoparticles concentration profile and its associated boundary layer thickness.

**Figure 20:**
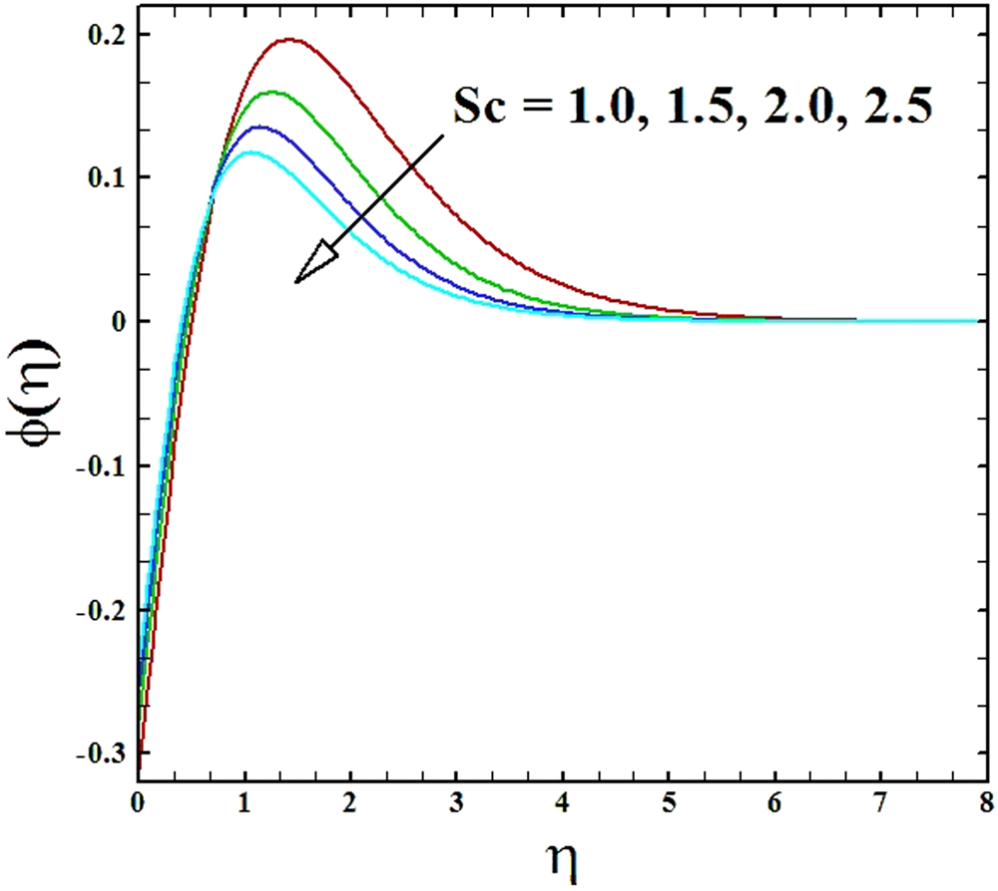
Behavior of ***ϕ***(***η***) versus escalating values of Schmidt number.

To explore the behavior of Schmidt number *Sc* on nanoparticles concentration profile Fig. 20 is plotted. We perceived that the nanoparticles concentration profile and its accompanying boundary layer thickness depreciates by the elevated values of Schmidt number.

## Final remarks

We numerically deliberated the effects of nonlinear radiation and heat generation/absorption on the axisymmetric MHD Carreau nanofluid flow with impact of chemical reaction over a radially stretching sheet by using MATLAB tool bvp4c. The effects of some pertaining non-dimensional physical parameters on involved distributions are also presented through the graphical illustrations. Tabulated numerically calculated values with requisite discussions are also included to the problem.

The following outcomes are observed after conducting the complete study:

- Velocity distribution is declining function of Weissenberg number and magnetic parameter.
- The thermal and nanoparticles concentration boundary layers are the incrementing functions of magnetic parameter, Weissenberg number, heat generation parameter, nonlinear radiation parameter, temperature ratio parameter and thermophoresis parameter.
- The nanoparticle concentration profile is diminishing function of Schmidt number, chemical reaction Brownian motion parameters.
- Skin friction coefficient diminishes with upsurge in magnetic parameter.
- Nonlinear radiation and heat generation parameters results the diminution of local Nusselt number while the temperature ratio parameter shows the antithesis result.
